# Spontaneous coronary artery dissection in a patient with hereditary polycystic kidney disease and a recent liver transplant: a case report

**DOI:** 10.1093/ehjcr/ytz216

**Published:** 2019-12-13

**Authors:** Ezther Verlaeckt, Laurens Van de Bruaene, Mathieu Coeman, Sofie Gevaert

**Affiliations:** 1 Department of Internal Medicine, Ghent University Hospital, Corneel Heymanslaan 10, 9000 Ghent, Belgium; 2 Department of Cardiology, Ghent University Hospital, Corneel Heymanslaan 10, 9000 Ghent, Belgium

**Keywords:** Spontaneous coronary dissection (SCAD), Autosomal dominant polycystic kidney disease (ADPKD), Acute coronary syndrome (ACS), Liver transplant, Corticosteroids, Case report

## Abstract

**Background:**

Spontaneous coronary artery dissection (SCAD) is an underestimated cause of acute coronary syndromes. A predisposing arteriopathy is often present and a stressor can sometimes be identified. Autosomal dominant polycystic kidney disease (ADPKD) is the most common hereditary kidney disorder; its associated arteriopathy has been described as a predisposing condition for SCAD.

**Case summary:**

A 44-year-old woman with ADPKD presented in the emergency room with recent onset thoracic pain radiating to the left arm at rest. She had undergone a recent liver transplant, for which she had received high-dose corticosteroids during 1 month. She was still taking tacrolimus and mycophenolate mofetil. She had no traditional risk factors but had experienced stress postoperatively. She was known with moderate chronic kidney disease. The initial electrocardiogram (ECG) was normal but high-sensitive troponin T was significantly elevated. Coronary angiography demonstrated diffuse narrowing of the distal left anterior descending artery with preserved flow, compatible with a SCAD Type 2 that was treated conservatively. However, under dual antiplatelet therapy (DAPT) with clopidogrel, the coronary dissection was progressive with new ischaemic ECG changes, further rise of troponins and development of apicoseptal hypokinesia. Because of the small vessel diameter and the preserved distal flow, conservative treatment was maintained. Clopidogrel was interrupted and the patient remained stable.

**Discussion:**

As SCAD remains an underestimated cause of myocardial infarction, clinicians should be aware of the possibility of SCAD in ADPKD patients with chest pain. This case report illustrates that the decision DAPT vs. aspirin should be individualized in these patients.

**Learning points**

- Spontaneous coronary artery dissection (SCAD) patients often have little or no traditional cardiovascular risk factors.
- Autosomal dominant polycystic kidney disease associated arteriopathy can predispose to SCAD.
- Corticosteroids can trigger the event.
- Treatment of SCAD is usually conservative because of the high probability of spontaneous healing, while intervention is reserved for cases with high-risk features like left main dissection, ongoing ischaemia, electrical, or haemodynamic instability.
- Prolonged inpatient monitoring (5–7 days) is recommended because of the risk of progression of the dissection.
- The need for dual antiplatelet therapy should be carefully evaluated in conservatively treated SCAD patients.

## Introduction

Spontaneous coronary artery dissection (SCAD) is defined as a spontaneous (non-traumatic, non-iatrogenic, and non-atherosclerotic) separation of the coronary artery wall by intramural haemorrhage resulting in myocardial ischaemia. It is elicited by an intimal tear or a spontaneous intramural haemorrhage. Patients can present with the whole spectrum of acute coronary syndromes (ACS). The prevalence in the general population has been underestimated as a result of underdiagnosis. Recently, the condition is gaining recognition and recent angiographic data report an incidence of SCAD in 1–4% of patients presenting with ACS.^1^^,^^2^ The aetiology of SCAD appears to be multifactorial but patients often have a predisposing condition and, in many cases, a precipitating trigger is recognized. Predisposing conditions include heritable connective tissue disorders, fibromuscular dysplasia, systemic inflammatory disease, and pregnancy-related hormonal changes, as they can cause weakening of the arterial wall. Emotional and extreme physical stress have been described as possible triggers, while hormonal therapy and corticosteroids have been identified as both predisposing factors and precipitating triggers (see Table 1).^3^

**Table 1:** Possible predisposing conditions and triggers for spontaneous coronary artery dissection

	Case patient
**Known predisposing conditions for SCAD**	
Inherited arteriopathy and connective tissue disorder	ADPKD
Systemic inflammatory disease	No evidence
Fibromuscular dysplasia	No evidence
Exogenous hormones	Was recently on high-dose steroid therapy (tapered 1 month before)
Pregnancy and multiparity	No (recent) pregnancy, one daughter
**Known triggers for SCAD**	
Extreme emotional stress	Present
Intense exercise	Not recent
Active hormonal therapy	Was recently on high-dose steroid therapy (tapered 1 month before)
Recreational drugs	None

Autosomal dominant polycystic kidney disease (ADPKD) is the most common hereditary kidney disorder. It is caused by mutations in PKD1 and -2 genes, coding for polycystin 1 and 2, proteins with an important role in the development and maintenance of the vascular system.^4^ Cystic malformation leads to progressive loss of kidney function over decades.^4^^,^^5^ It is a systemic disorder with polycystic liver disease being the most common extrarenal manifestation.^4^ Intracranial aneurysms are the most common vascular manifestation.^5^ Common cardiac manifestations include mitral valve prolapse and aortic insufficiency.^4^

## Timeline

**Table:** 

Day	Events
1	Consultation abdominal surgery after recent liver transplant: complaints of intermittent chest pain, cardiological workup planned.
4	Admission to the emergency room: persisting heavy chest pain.
	Electrocardiogram (ECG) showed a normal sinus rhythm without ST segment or T-wave abnormalities.
	Blood samples revealed elevated cardiac troponins T (53 pg/mL) but without evolution.
	Transthoracic echocardiogram (TTE) showed a non-dilated ventricle wild mild septal hypertrophy, normal regional, and global left ventricular function without significant valvulopathy.
	Coronary angiography was performed and showed a diffuse stenosis of the distal left anterior descending artery (LAD) compatible with a spontaneous coronary artery dissection. Conservative treatment with dual antiplatelet therapy, low-dose statin, and beta-blocker was started.
	The patient was admitted to the intensive cardiac care unit for further evaluation.
6	New episode of chest pain: ECG showed diffuse ST segment and repolarization abnormalities.
	A new TTE showed apicoseptal hypokinesia.
	A new coronary angiography showed a more proximal and prominent dissection of the LAD (75–95% stenosis) with preserved distal flow. No percutaneous coronary intervention because of small vessel size.
	Transdermal nitrates were associated, clopidogrel was interrupted and a small dose of angiotensin-converting-enzyme inhibition was initiated. Afterwards, the patient remained pain free.
7	Prolonged monitoring reveals a single asymptomatic episode of non-sustained ventricular tachycardia.
10	Discharge under medical treatment with close follow-up.
33	Follow-up outpatient visit, no problems. Initiation of cardiac rehabilitation.

## Case presentation

**Figure 1:**
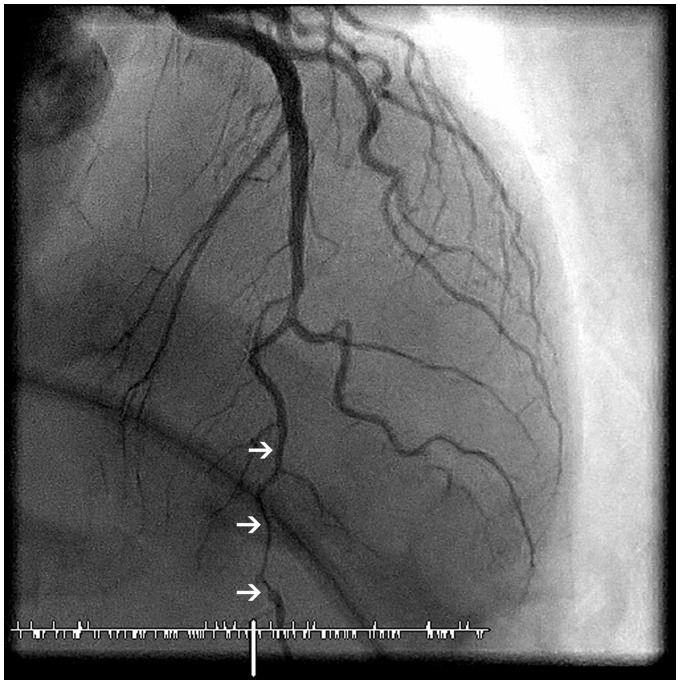
Coronary angiogram Day 0: apical 0°, cranial 35° demonstrating diffuse stenosis of the mid and distal LAD with ‘stick insect’ aspect (arrows).

**Figure 2:**
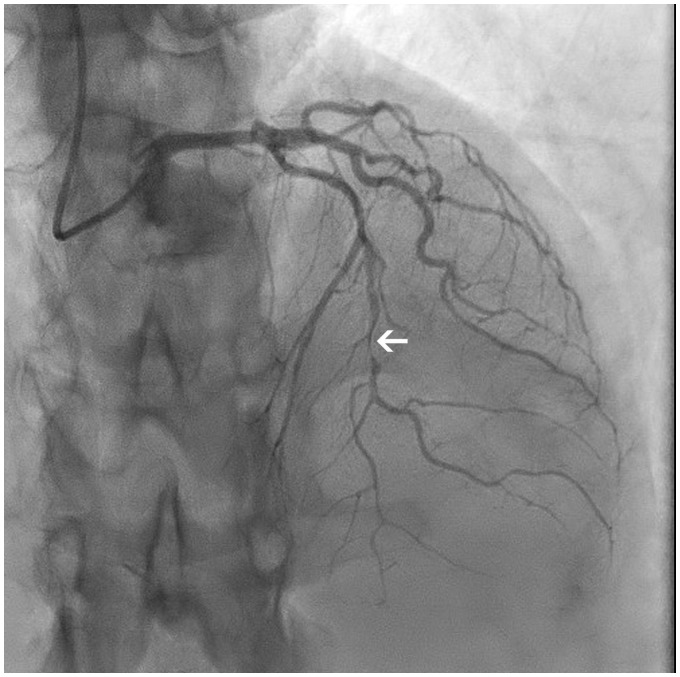
Coronary angiogram Day 4: apical 0°, cranial 38° demonstrating more proximal and prominent dissection (arrow).

A 44-year-old woman was referred by her gastrointestinal surgeon to the emergency room (ER) because of multiple episodes of chest pain. She was, like her mother, known with ADPKD as well as polycystic liver disease, without (intracranial) aneurysms. Two months earlier, a liver transplantation had been performed because of dyspnoea caused by the liver cysts. She was under treatment with antirejection medication (tacrolimus, mycophenolate mofetil, and steroids that were tapered recently). She did not smoke and there were no other cardiovascular risk factors but stress. The chest pain started during sleep, radiated to the left arm and subsided spontaneously after 5 min. The electrocardiogram (ECG) in the ER showed a normal sinus rhythm without ST segment or T-wave abnormalities. Patient was afebrile, with a blood pressure of 137/74 mmHg and a regular pulse of 77 b.p.m. On auscultation heart sounds were normal and lungs were clear. There was no peripheral oedema and peripheral pulsations were normal. Chest X-ray was normal. Blood samples showed a chronic but stable kidney disease (eGFR 36 mL/min/1.73 m^2^), absence of inflammation or anaemia. High-sensitive troponin T-level was significantly elevated (53 pg/mL) but showed no significant evolution after 3 h. A transthoracic echocardiogram (TTE) demonstrated a non-dilated ventricle (end-diastolic diameter 41 mm) with mild septal hypertrophy (14 mm), normal regional contractility, a preserved left ventricular (LV) systolic function and absence of significant valvulopathy. A coronary angiography was performed and showed diffuse narrowing of the distal left anterior descending artery (LAD) with ‘stick insect’ aspect, compatible with a SCAD Type 2 with preserved distal flow^6^ (*Figure 1*). The patient was admitted to the intensive cardiac care unit for further observation and treatment. Dual antiplatelet therapy (DAPT) with clopidogrel and aspirin was started, as well as pravastatin and a low dose of bisoprolol. During the following days, the troponin level steadily rose with stable kidney function. Three days later, chest pain redeveloped for which IV nitrates were associated but these were quickly interrupted because of headache. The ECG was progressively abnormal with diffuse biphasic T waves in the anterior leads (V2–5). A repeat TTE now showed apicoseptal hypokinesia. A new coronary angiography showed a more proximal and prominent dissection of the mid and distal LAD (75–95% stenosis), with preserved distal flow (*Figure 2*). Transdermal nitrates were associated after which the pain subsided. Clopidogrel was interrupted because of the progression under DAPT and a small dose of angiotensin-converting-enzyme (ACE)-inhibition was initiated because of the depressed LV function. Prolonged monitoring revealed a single episode of non-sustained ventricular tachycardia. The patient remained asymptomatic and the rest of the stay was uneventful. She was discharged after 10 days and was still doing well at follow-up after 1 month with cardiac enzymes that returned to baseline levels. Cardiac rehabilitation, including psychological support, was initiated.

## Discussion

We report a SCAD in an ADPKD patient, an association of which only eight cases have been described in the literature.^7^^,^^8^ A recent nationwide retrospective analysis of 66 360 SCAD cases, however, identified 60 patients (0.09%) with ADPKD.^9^ The association between SCAD and inherited arteriopathy and connective tissue disorder is known.^1–3^ Autosomal dominant polycystic kidney disease is a known predisposing factor for vascular abnormalities, especially cerebral aneurysms.^4^ Our patient did not have intracranial aneurysms or vascular abnormalities in the thorax or abdomen. The recent treatment with corticosteroids and the associated stress may have triggered the SCAD and to our knowledge, this is the first case of SCAD in a recently transplanted ADPKD patient.

Most (>90%) SCAD patients are women and typically have a low cardiovascular risk profile, which may cause delayed diagnosis.^1^^,^^3^ However, thanks to increasing awareness and the introduction of high-sensitive troponins diagnosis has improved.^10^

The management of SCAD differs from that of atherosclerotic coronary artery disease.^1–3^ In our case, no other classical risk factors but chronic kidney disease and emotional stress were identified. She was on steroid antirejection therapy the month before, which is a possible precipitating factor for SCAD.^1–3^

Coronary angiography is the gold standard for the diagnosis of SCAD with intracoronary imaging (optical coherence tomography or intravascular ultrasound) being preserved for uncertain diagnosis. The commonly used angiographic classification consists of three types. Type 1 represents the pathognomonic form with multiple radiolucent lumens and extraluminal contrast staining. Type 2, the commonest type, refers to a diffuse stenosis of varying length (usually >20 mm), while a Type 3 SCAD is a focal or tubular and usually short (<20 mm) stenosis that mimics atherosclerosis. In addition, a Type 4 presentation characterized by a total occlusion, usually of a distal vessel is described in the European position paper on SCAD.^1^ Some familiarity with these angiographic appearances is needed to recognize SCAD but further intracoronary imaging can be necessary, especially in Type 3 presentations. The LAD is the most affected vessel and there is a predisposition for distal segments, as seen in this case.^1^^,^^3^

There is increasing evidence of angiographic healing with medical treatment and percutaneous intervention is associated with a high complication rate.^11^ Therefore, medical treatment is preferred over revascularization in case of preserved flow and absence of high-risk features like left main dissection, ongoing ischaemia, electrical, or haemodynamic instability.^1–3^^,^^12^ In this case, angina was controlled medically, furthermore, percutaneous intervention was not possible because of the small size of the vessel.

There is little evidence for medical treatment of SCAD, current recommendations are based upon expert opinion. Dual antiplatelet therapy with aspirin and clopidogrel was started in this case, as recommended by most experts (1–12 months) because of evidence that high-grade stenosis can be associated with true luminal thrombus in SCAD patients. However, in contrast with a classic ACS, clopidogrel is preferred over newer P2Y12 inhibitors like ticagrelor and prasugrel because of lack of data and their higher associated bleeding risk. Here, clopidogrel was interrupted because of the progression of the dissection, indicating that the need for DAPT should be evaluated on a patient by patient basis. Given the LV impairment, beta-blockers, and ACE-inhibitors were indicated in this patient.^1–3^ Statins are not routinely recommended since the aetiology of SCAD is not atherosclerotic. Furthermore, a small retrospective cohort demonstrated a higher recurrence rate in statin users, therefore, their use should be reserved for patients with conventional indications for statins.^1–3^

In 5–10% of SCAD patients on medical treatment, extension of dissection occurs in the early phase, as in our case. Therefore prolonged inpatient monitoring (5–7 days) is recommended in SCAD patients.^1–3^ Despite a favourable 30 days survival, 30-day major adverse cardiovascular events rate is high (9%).^2^^,^^13^

Recurrent SCAD can complicate the long-term course and has been reported to be as high as 30% in older series. Unfortunately, there is no treatment strategy that has shown to reduce this risk. In a recent prospective cohort study, this risk was substantially lower (10.4% over a 3-year time course), and therapy with beta-blockade seemed to reduce recurrence risk, probably by lowering the coronary arterial wall stress through decrease in myocardial contractility and blood pressure.^14^

It is very important to acknowledge the impact of SCAD on psychological well-being. Rates of depression and anxiety in SCAD patients are similar to those of non-SCAD myocardial infarction patients.^15^ Therefore, specific rehabilitation programmes including psychological support are strongly recommended.^16^ Patient groups active on social media, such as Beat SCAD-UK and SCAD Alliance, aim to support SCAD patients and their families and raise awareness of SCAD.

## Conclusion

Autosomal dominant polycystic kidney disease associated arteriopathy predisposes to SCAD and high doses of corticosteroids, given in the transplant setting, may trigger the event. Caregivers should be aware of this association when evaluating ADPKD patients with chest pain. Most cases can be managed conservatively with medical management. Dual antiplatelet therapy with aspirin and clopidogrel is recommended by experts but should be evaluated on an individual basis.

## Lead author biography

**Figure:**
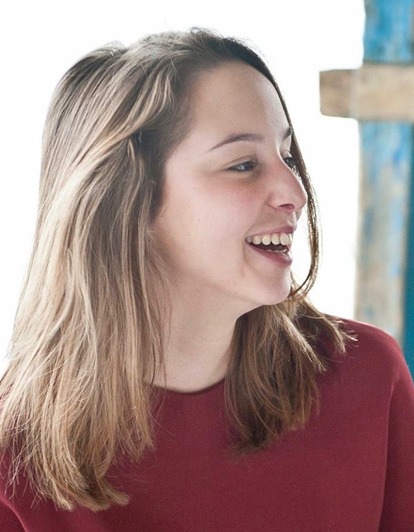


Ezther Verlaeckt received her medical degree at Ghent University in 2019. She then started her residency in internal medicine.

## Supplementary material

Supplementary material is available at *European Heart Journal - Case Reports* online.

**Slide sets:** A fully edited slide set detailing this case and suitable for local presentation is available online as Supplementary data.

**Consent:** The author/s confirm that written consent for submission and publication of this case report including image(s) and associated text has been obtained from the patient in line with COPE guidance.

**Conflict of interest:** none declared.

## Supplementary Material

ytz216_Supplementary_Slide_Set
