# Supplementary material for: Spontaneous coronary artery dissection in a patient with hereditary polycystic kidney disease and a recent liver transplant: a case report
Source: Eur Heart J Case Rep. 2019 Dec 13;3(4):1–5. doi: 10.1093/ehjcr/ytz216 (PMC6939822; doi:10.1093/ehjcr/ytz216)
Supplement: ytz216_Supplementary_Slide_Set [file ytz216_supplementary_slide_set.pptx]

## Slide 1
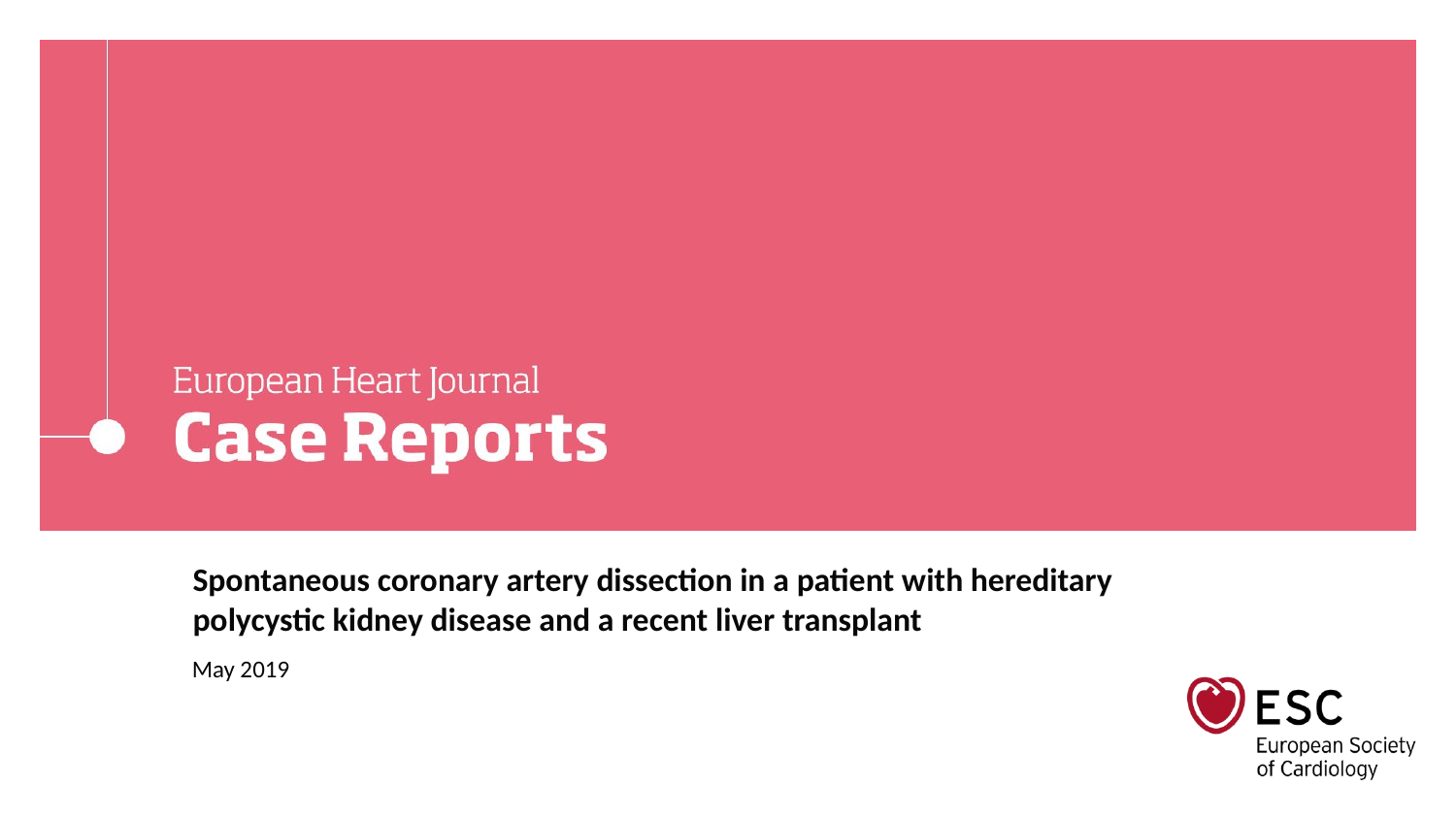

# Spontaneous coronary artery dissection in a patient with hereditary polycystic kidney disease and a recent liver transplant
May 2019

## Slide 2
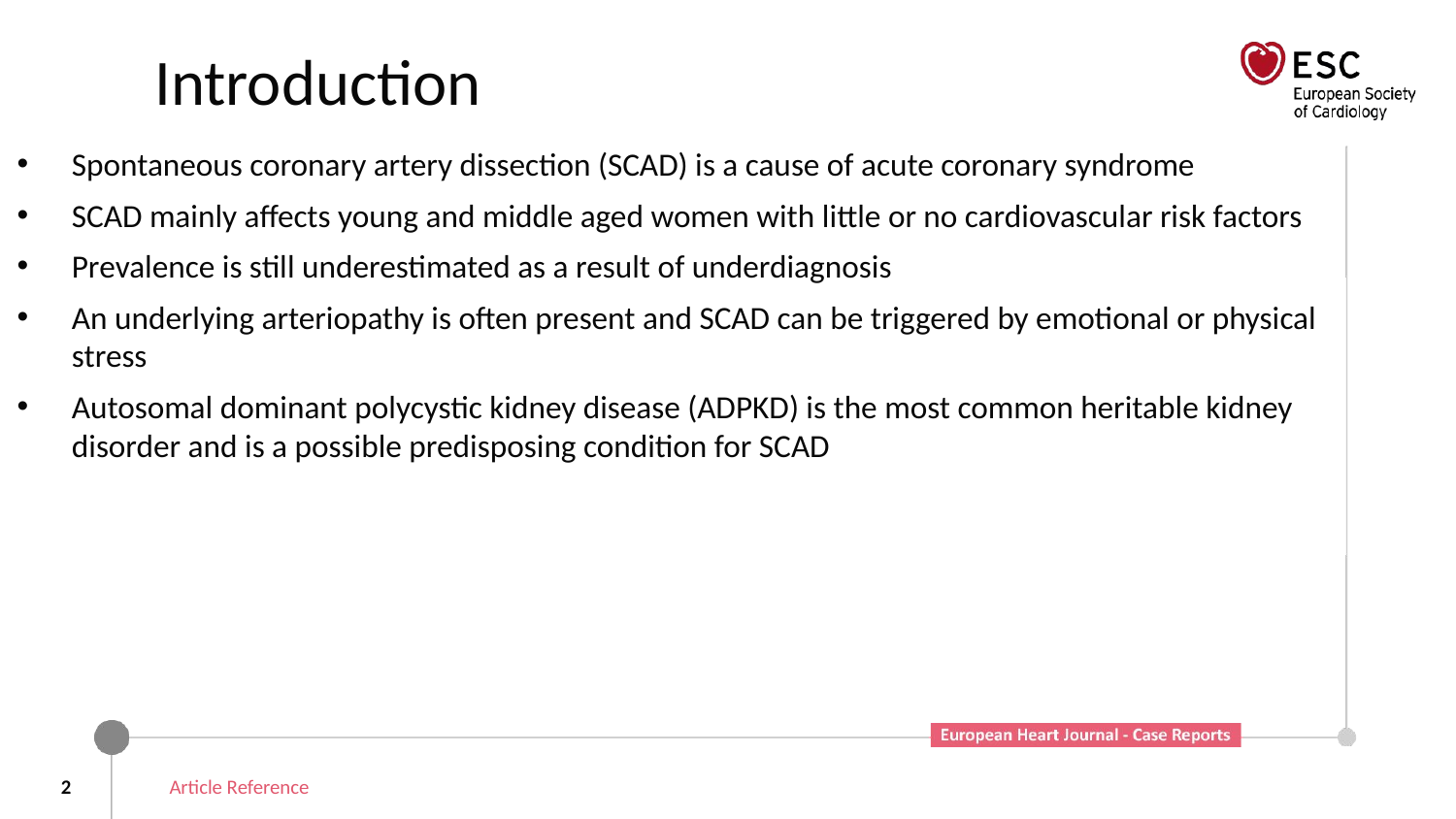

# Introduction
Spontaneous coronary artery dissection (SCAD) is a cause of acute coronary syndrome
SCAD mainly affects young and middle aged women with little or no cardiovascular risk factors
Prevalence is still underestimated as a result of underdiagnosis
An underlying arteriopathy is often present and SCAD can be triggered by emotional or physical stress
Autosomal dominant polycystic kidney disease (ADPKD) is the most common heritable kidney disorder and is a possible predisposing condition for SCAD
2
Article Reference

## Slide 3
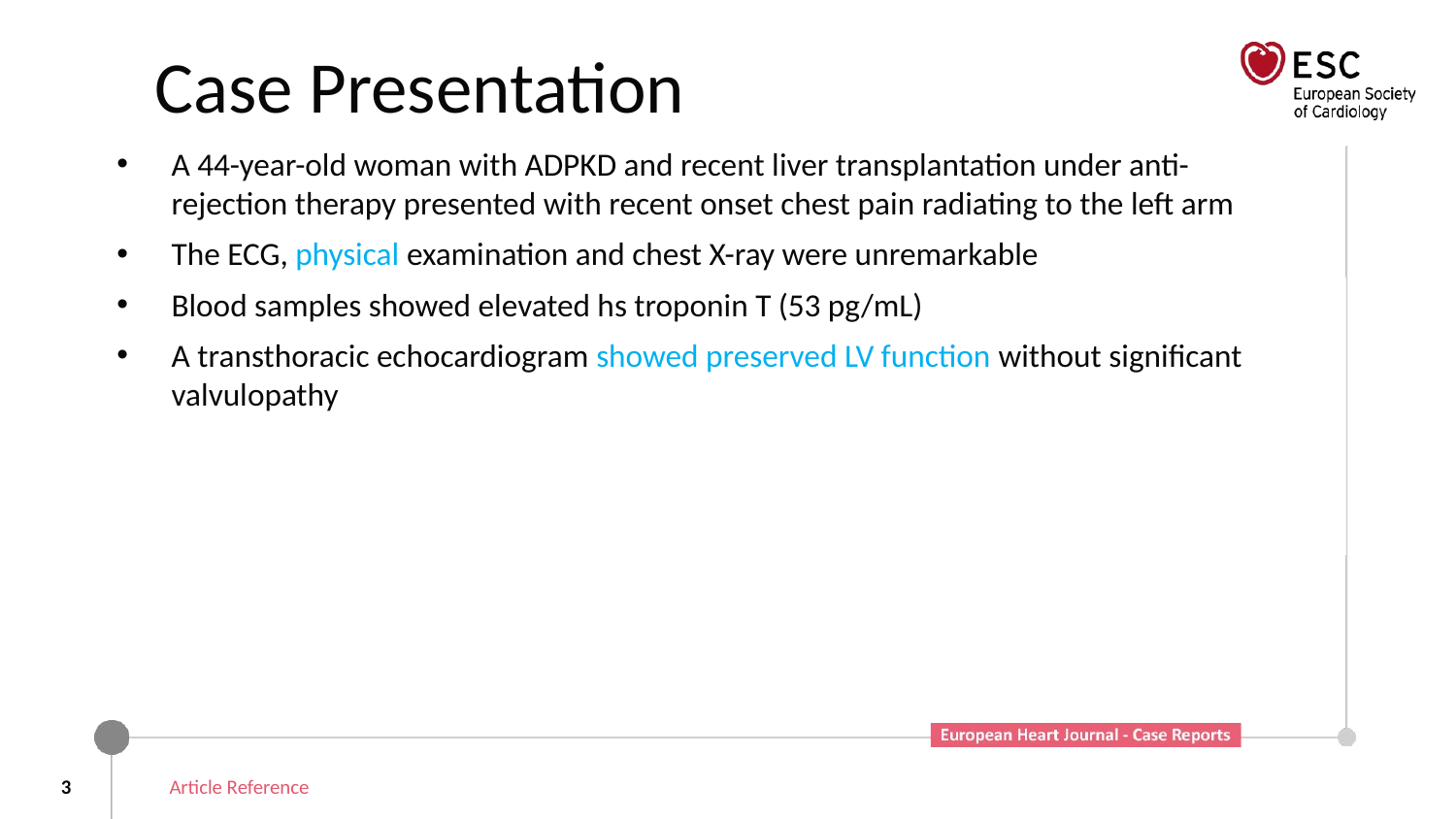

# Case Presentation
A 44-year-old woman with ADPKD and recent liver transplantation under anti-rejection therapy presented with recent onset chest pain radiating to the left arm
The ECG, physical examination and chest X-ray were unremarkable
Blood samples showed elevated hs troponin T (53 pg/mL)
A transthoracic echocardiogram showed preserved LV function without significant valvulopathy
3
Article Reference

## Slide 4
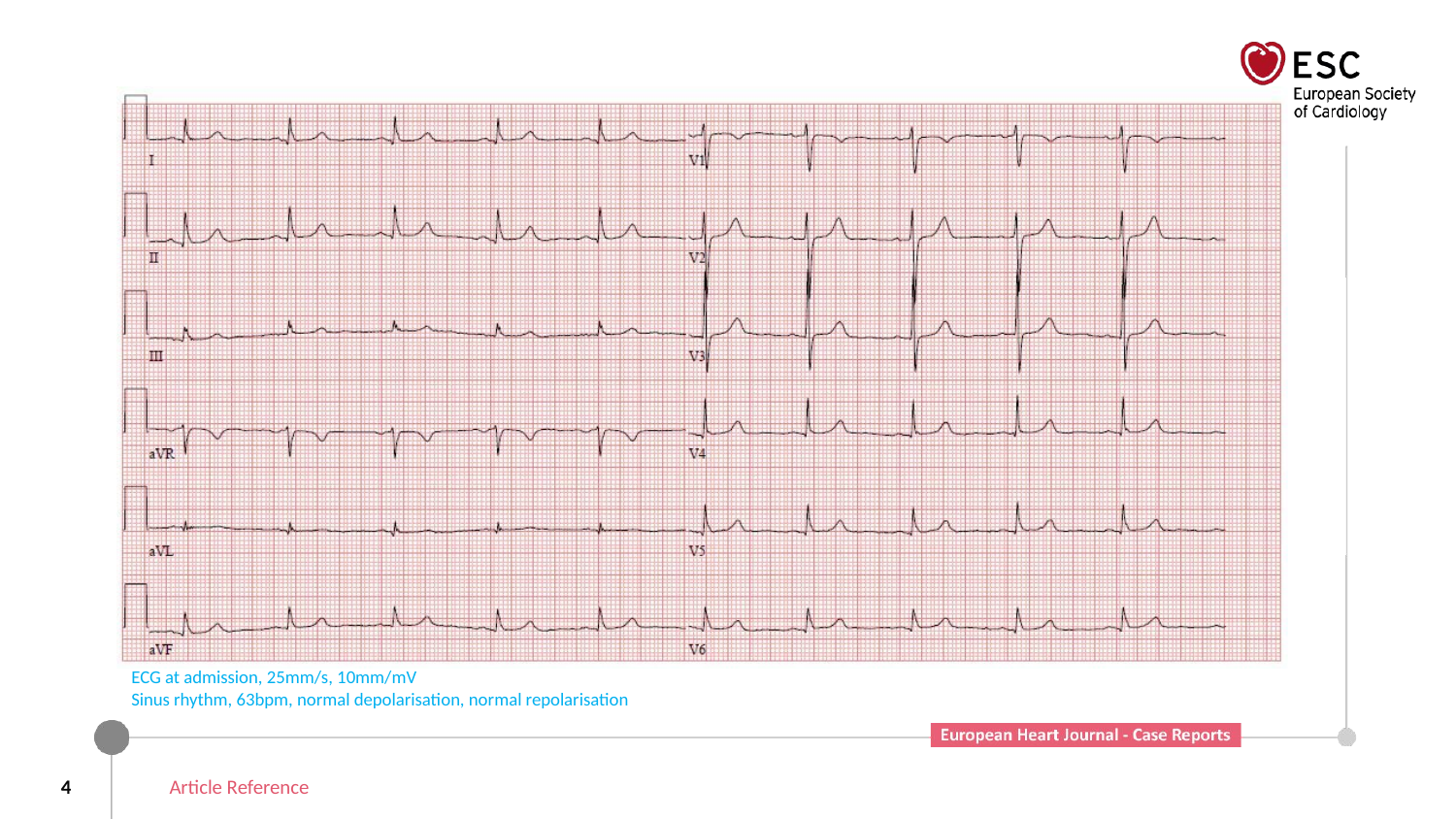

#
ECG at admission, 25mm/s, 10mm/mV
Sinus rhythm, 63bpm, normal depolarisation, normal repolarisation
4
Article Reference

## Slide 5
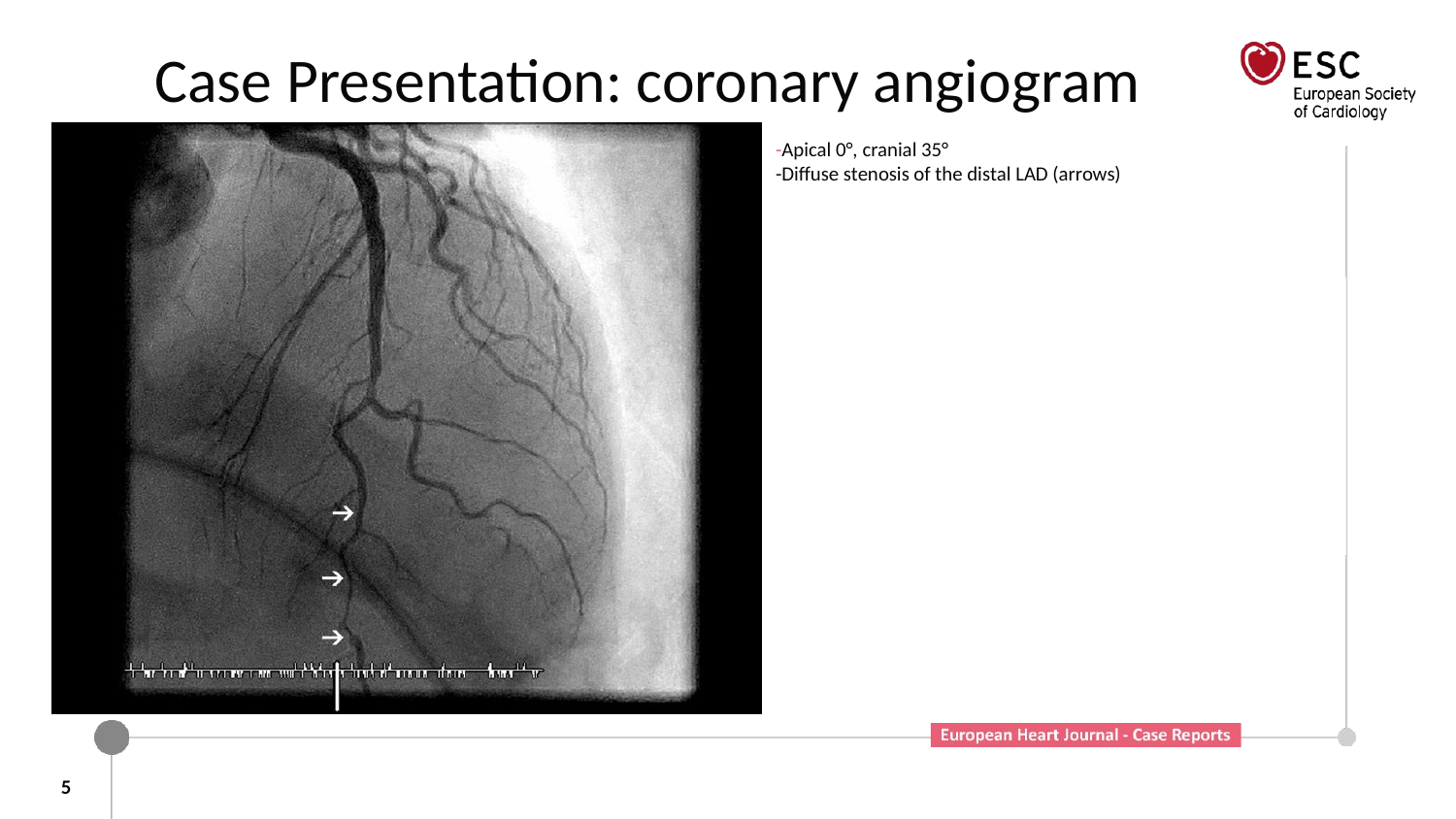

# Case Presentation: coronary angiogram
-Apical 0°, cranial 35°
-Diffuse stenosis of the distal LAD (arrows)
5

## Slide 6
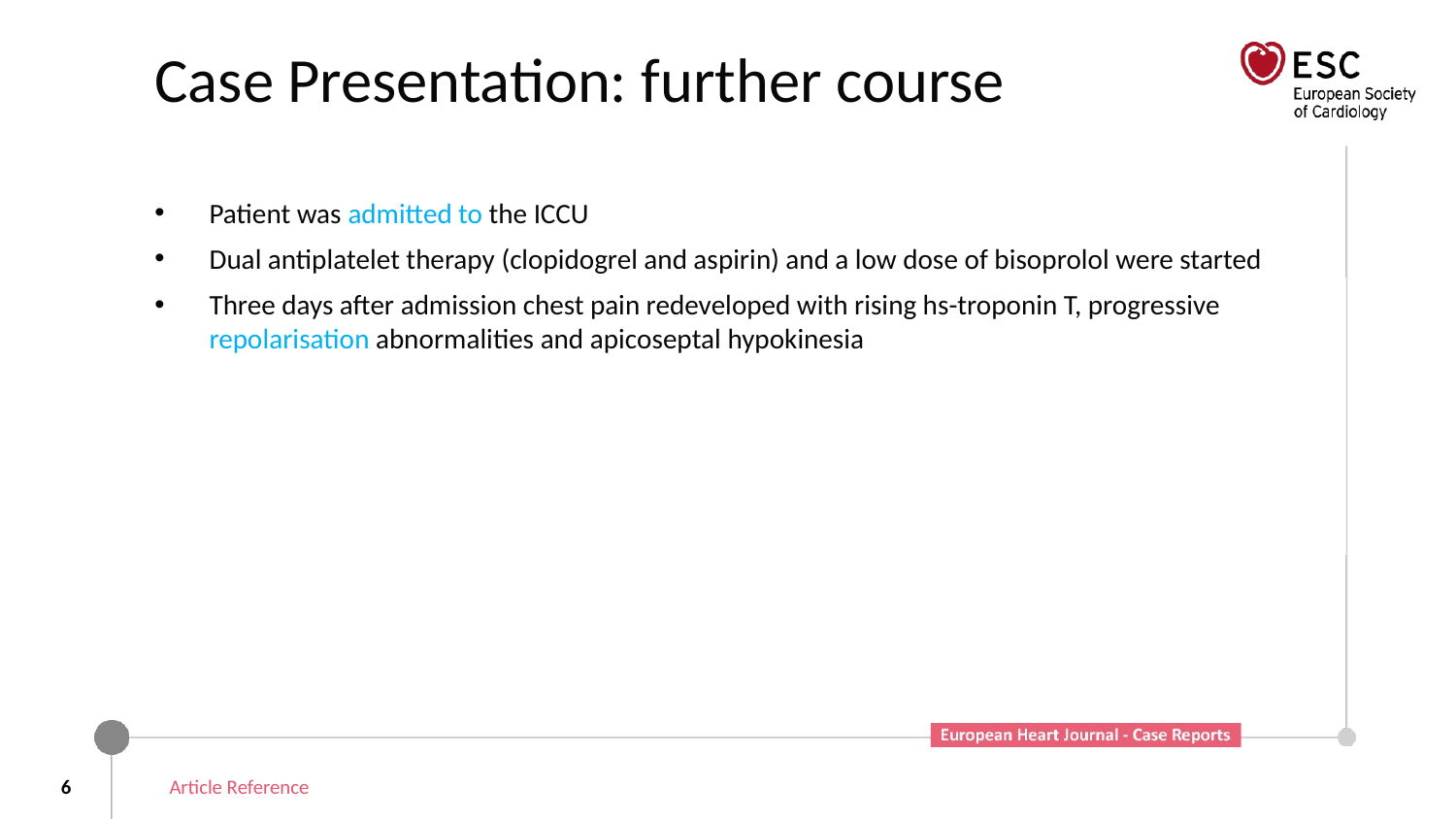

# Case Presentation: further course
Patient was admitted to the ICCU
Dual antiplatelet therapy (clopidogrel and aspirin) and a low dose of bisoprolol were started
Three days after admission chest pain redeveloped with rising hs-troponin T, progressive repolarisation abnormalities and apicoseptal hypokinesia
6
Article Reference

## Slide 7
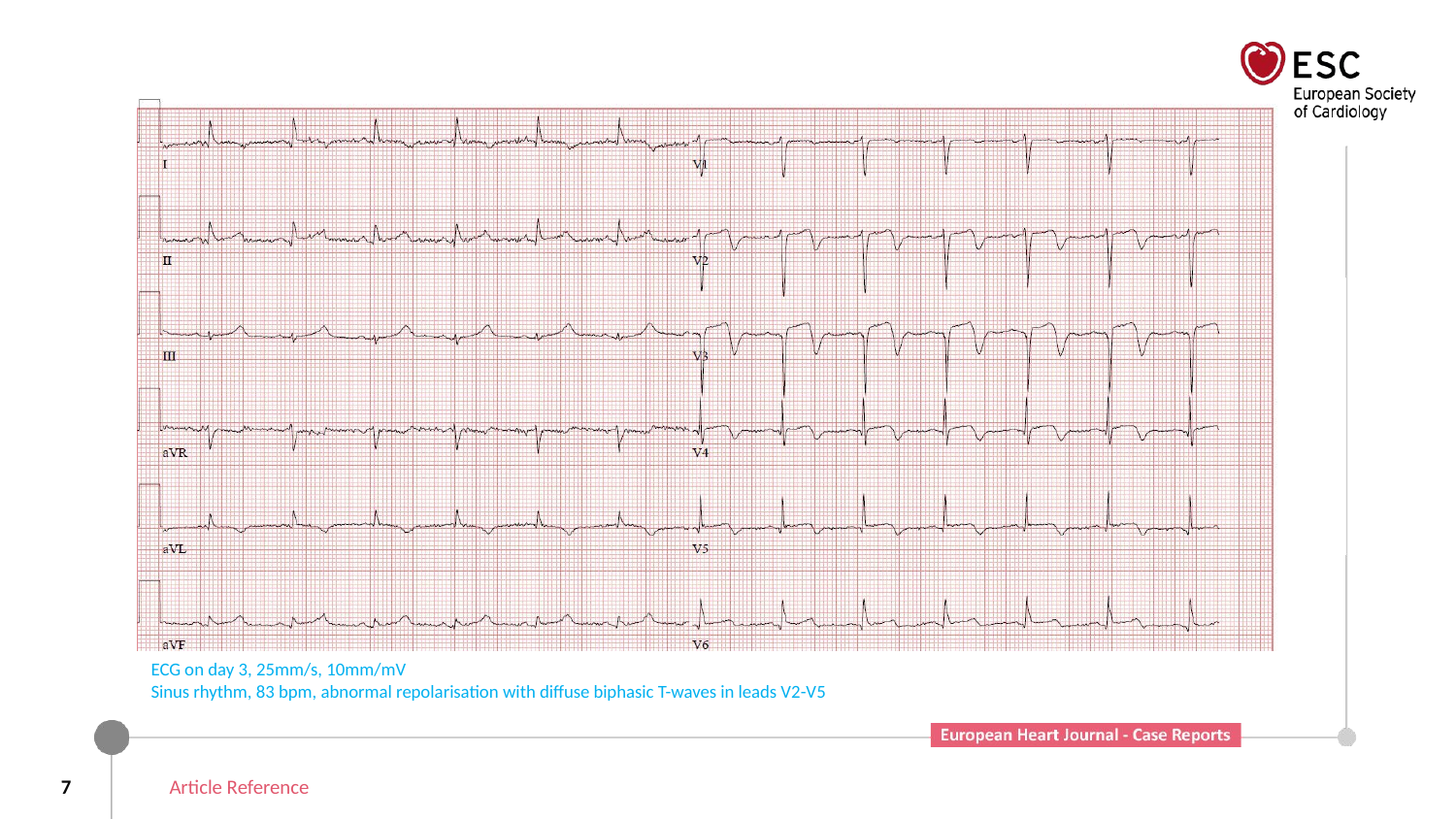

#
ECG on day 3, 25mm/s, 10mm/mV
Sinus rhythm, 83 bpm, abnormal repolarisation with diffuse biphasic T-waves in leads V2-V5
7
Article Reference

## Slide 8
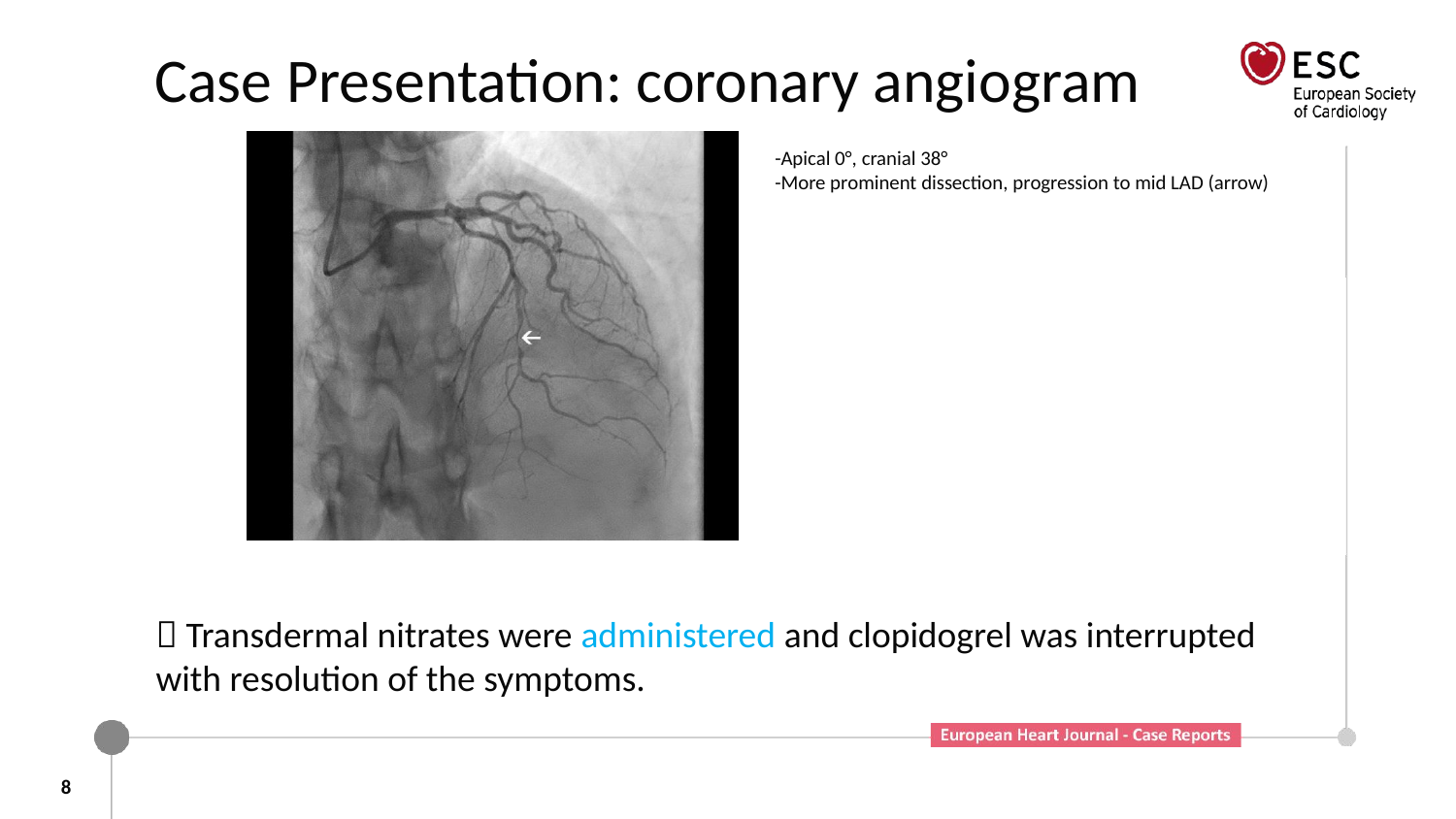

# Case Presentation: coronary angiogram
-Apical 0°, cranial 38°
-More prominent dissection, progression to mid LAD (arrow)
 Transdermal nitrates were administered and clopidogrel was interrupted with resolution of the symptoms.
8

## Slide 9
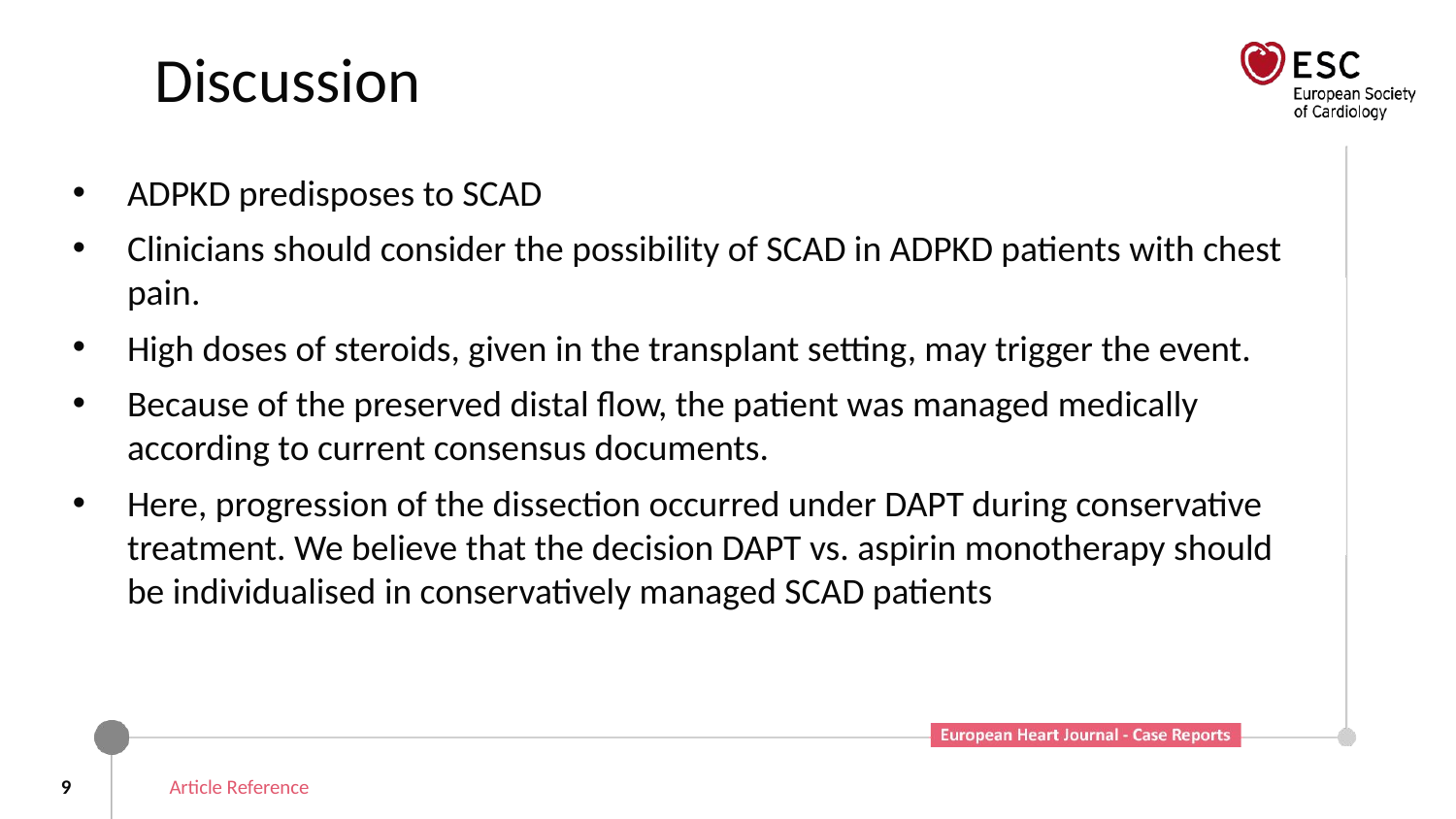

# Discussion
ADPKD predisposes to SCAD
Clinicians should consider the possibility of SCAD in ADPKD patients with chest pain.
High doses of steroids, given in the transplant setting, may trigger the event.
Because of the preserved distal flow, the patient was managed medically according to current consensus documents.
Here, progression of the dissection occurred under DAPT during conservative treatment. We believe that the decision DAPT vs. aspirin monotherapy should be individualised in conservatively managed SCAD patients
9
Article Reference

## Slide 10
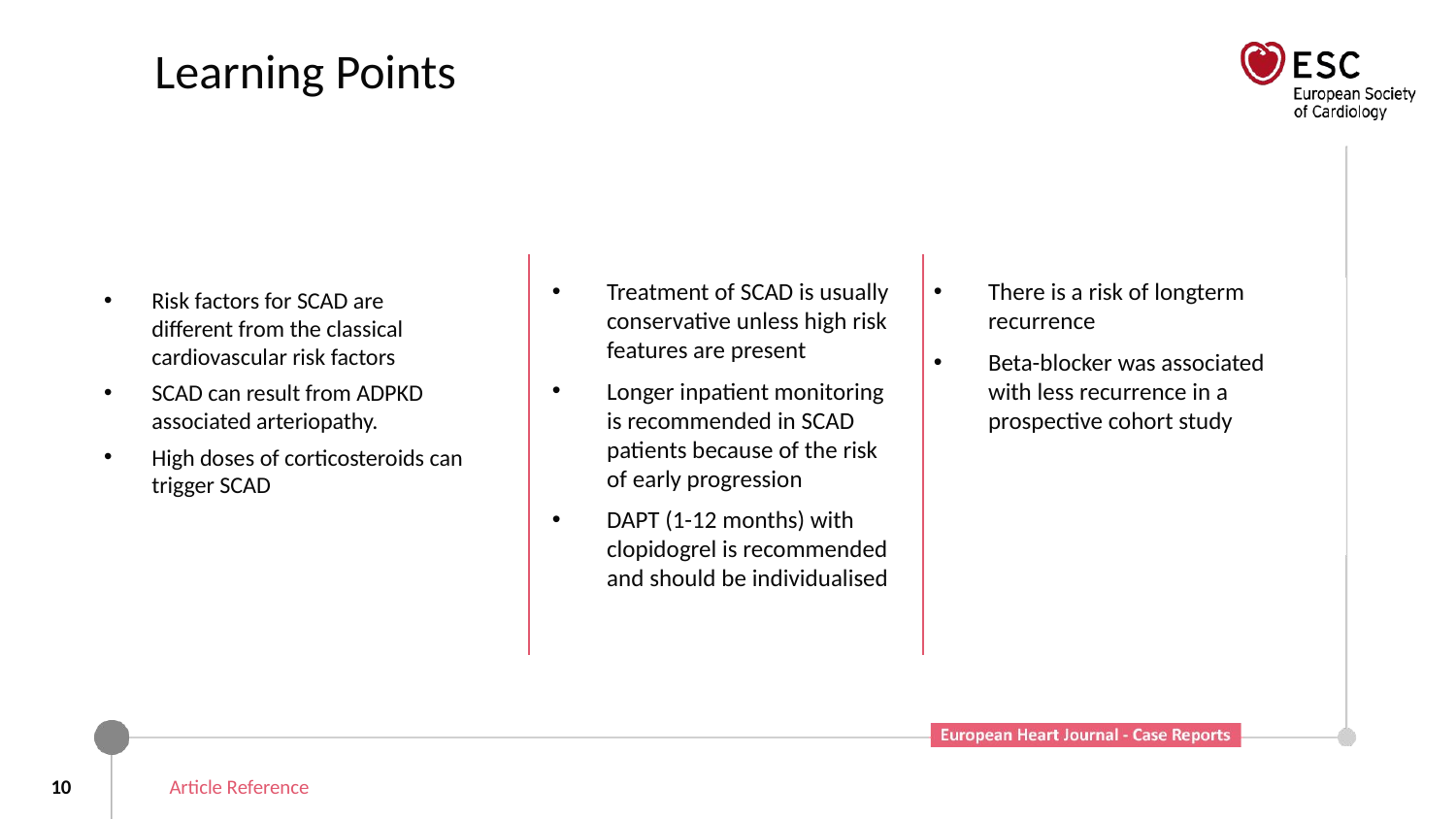

# Learning Points
Treatment of SCAD is usually conservative unless high risk features are present
Longer inpatient monitoring is recommended in SCAD patients because of the risk of early progression
DAPT (1-12 months) with clopidogrel is recommended and should be individualised
There is a risk of longterm recurrence
Beta-blocker was associated with less recurrence in a prospective cohort study
Risk factors for SCAD are different from the classical cardiovascular risk factors
SCAD can result from ADPKD associated arteriopathy.
High doses of corticosteroids can trigger SCAD
10
Article Reference

## Slide 11
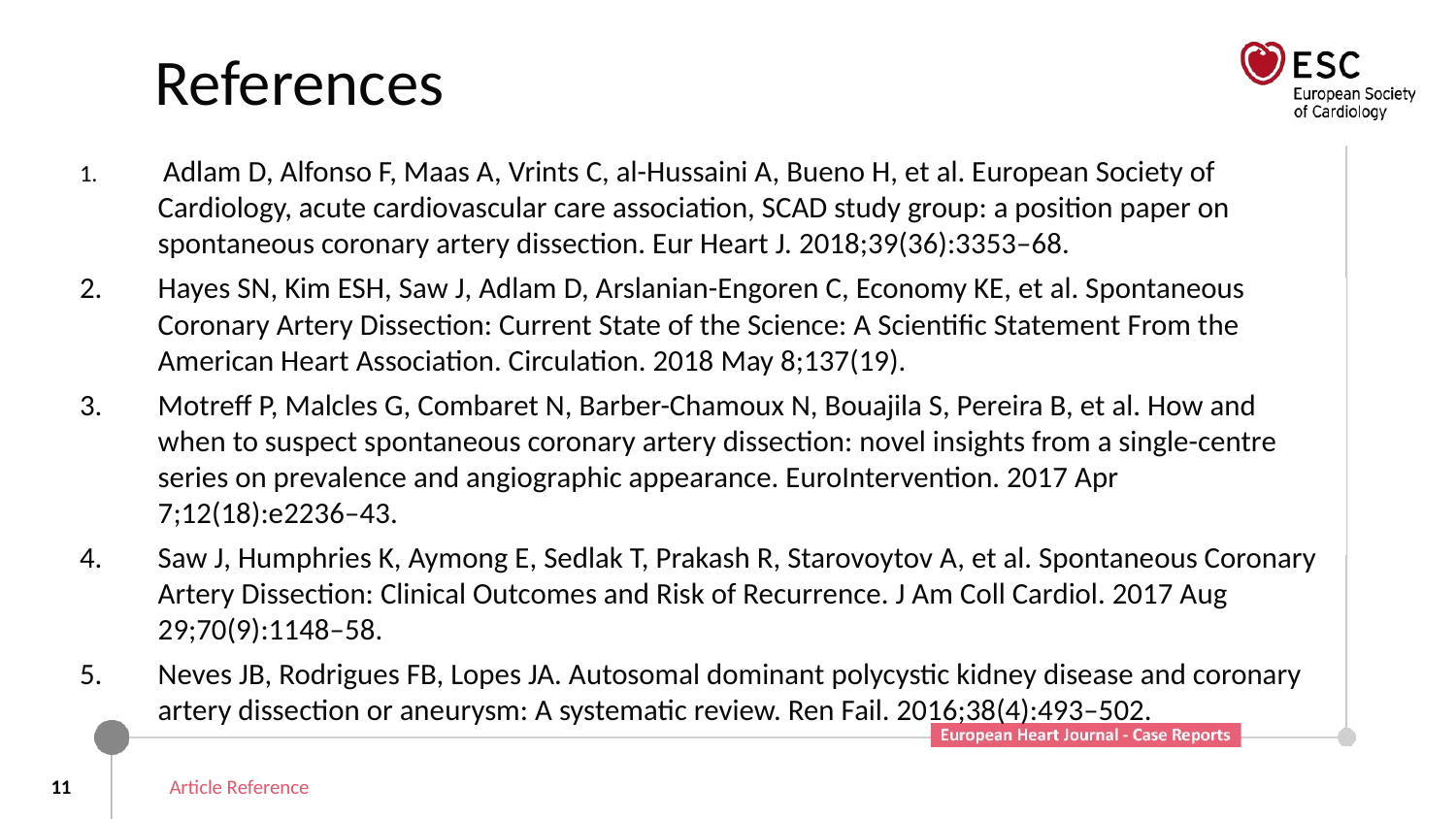

# References
 Adlam D, Alfonso F, Maas A, Vrints C, al-Hussaini A, Bueno H, et al. European Society of Cardiology, acute cardiovascular care association, SCAD study group: a position paper on spontaneous coronary artery dissection. Eur Heart J. 2018;39(36):3353–68.
Hayes SN, Kim ESH, Saw J, Adlam D, Arslanian-Engoren C, Economy KE, et al. Spontaneous Coronary Artery Dissection: Current State of the Science: A Scientific Statement From the American Heart Association. Circulation. 2018 May 8;137(19).
Motreff P, Malcles G, Combaret N, Barber-Chamoux N, Bouajila S, Pereira B, et al. How and when to suspect spontaneous coronary artery dissection: novel insights from a single-centre series on prevalence and angiographic appearance. EuroIntervention. 2017 Apr 7;12(18):e2236–43.
Saw J, Humphries K, Aymong E, Sedlak T, Prakash R, Starovoytov A, et al. Spontaneous Coronary Artery Dissection: Clinical Outcomes and Risk of Recurrence. J Am Coll Cardiol. 2017 Aug 29;70(9):1148–58.
Neves JB, Rodrigues FB, Lopes JA. Autosomal dominant polycystic kidney disease and coronary artery dissection or aneurysm: A systematic review. Ren Fail. 2016;38(4):493–502.
11
Article Reference
